# Supplementary material for: A Likelihood Approach for Real-Time Calibration of Stochastic Compartmental Epidemic Models
Source: PLoS Comput Biol. 2017 Jan 17;13(1):e1005257. doi: 10.1371/journal.pcbi.1005257 (PMC5240920; doi:10.1371/journal.pcbi.1005257)
Supplement: S1 File — (TAR.GZ) [file pcbi.1005257.s014.tar.gz › HSPH_Online-SI-Revision/Additions/EnKF08/n100-extreme-SEITR/n100-extreme-SEITR_EnKF2_table4.pdf]

1	newly 3 weeks spec	1057.	2217.63	{259., 6440.}	147.596	0.415691	newly 3 weeks cumul	5452.	15 415.5	{7731., 40 738.}	190.991	0.427029	newly 5 weeks spec	382.	470.09	{13., 746.}	98.0759	0.609864	newly 5 weeks cumul	6457.	16 919.8	{8100., 47 894.}	170.73	0.393166
2	newly 3 weeks spec	909.	1212.02	{0., 1665.}	144.011	2.13905	newly 3 weeks cumul	5060.	17 607.9	{3104., 46 426.}	264.877	0.405161	newly 5 weeks spec	300.	568.03	{0., 817.}	202.943	1.94089	newly 5 weeks cumul	5879.	18 998.5	{3251., 61 719.}	238.576	0.378219
3	newly 3 weeks spec	1244.	1214.2	{117., 2402.}	81.3296	0.434064	newly 3 weeks cumul	6664.	6673.04	{711., 19 128.}	74.7089	0.359175	newly 5 weeks spec	463.	523.71	{46., 795.}	97.6134	0.556568	newly 5 weeks cumul	7872.	7976.54	{1024., 20 808.}	76.0191	0.358159
4	newly 3 weeks spec	1162.	1616.14	{43., 3191.}	108.554	0.559328	newly 3 weeks cumul	6474.	13 412.2	{2136., 33 484.}	135.021	0.333108	newly 5 weeks spec	412.	649.71	{0., 883.}	152.57	0.953378	newly 5 weeks cumul	7613.	15 062.3	{2359., 33 498.}	129.356	0.340007
5	newly 3 weeks spec	334.	698.25	{79., 1123.}	151.195	0.355789	newly 3 weeks cumul	2304.	4099.23	{1102., 6428.}	98.806	0.240642	newly 5 weeks spec	78.	309.	{3., 446.}	353.179	0.618696	newly 5 weeks cumul	2553.	4864.47	{1470., 7771.}	111.234	0.249719
6	newly 3 weeks spec	210.	3360.33	{82., 14 064.}	1526.22	0.629576	newly 3 weeks cumul	1688.	19 068.4	{2136., 63 350.}	1034.27	0.675333	newly 5 weeks spec	40.	716.23	{3., 1415.}	1751.28	0.751145	newly 5 weeks cumul	1825.	21 395.4	{2151., 74 778.}	1077.13	0.669703
7	newly 3 weeks spec	1990.	2437.94	{348., 5482.}	83.1417	0.365179	newly 3 weeks cumul	8888.	19 883.4	{8100., 48 406.}	131.207	0.265334	newly 5 weeks spec	982.	423.72	{16., 628.}	78.4644	0.815609	newly 5 weeks cumul	11 272.	21 310.5	{8571., 58 304.}	103.991	0.233734
8	newly 3 weeks spec	1262.	3470.18	{0., 12 661.}	278.296	2.2452	newly 3 weeks cumul	6258.	31 439.2	{5219., 88 188.}	415.808	0.579844	newly 5 weeks spec	510.	2202.04	{0., 11 186.}	440.169	2.1632	newly 5 weeks cumul	7632.	36 405.6	{6419., 101 405.}	389.72	0.558709
9	newly 3 weeks spec	351.	959.34	{176., 2033.}	197.869	0.360594	newly 3 weeks cumul	2373.	7423.7	{1756., 16 280.}	226.734	0.299272	newly 5 weeks spec	96.	351.88	{25., 554.}	308.292	0.5516	newly 5 weeks cumul	2626.	8333.07	{2234., 17 599.}	227.457	0.297838
10	newly 3 weeks spec	2417.	2532.51	{0., 8046.}	105.909	1.10148	newly 3 weeks cumul	10 500.	19 166.8	{1484., 70 368.}	131.855	0.38236	newly 5 weeks spec	1167.	680.6	{0., 1466.}	78.8963	1.26026	newly 5 weeks cumul	13 414.	21 157.8	{1728., 75 459.}	117.19	0.397286
11	newly 3 weeks spec	2013.	3525.76	{122., 9030.}	170.256	0.653782	newly 3 weeks cumul	9231.	13 648.1	{698., 30 147.}	129.636	0.530558	newly 5 weeks spec	1003.	1858.13	{18., 7386.}	192.484	0.847152	newly 5 weeks cumul	11 702.	18 114.6	{778., 53 145.}	140.782	0.56815
12	newly 3 weeks spec	1233.	3951.29	{160., 12 162.}	263.115	0.465097	newly 3 weeks cumul	6317.	20 729.5	{3659., 54 624.}	243.154	0.373629	newly 5 weeks spec	542.	2019.89	{10., 7692.}	338.633	0.771462	newly 5 weeks cumul	7681.	25 575.3	{4252., 79 811.}	249.919	0.377657
13	newly 3 weeks spec	1573.	2500.03	{282., 7228.}	117.434	0.374938	newly 3 weeks cumul	7548.	20 657.8	{3451., 50 779.}	190.168	0.332614	newly 5 weeks spec	706.	648.38	{37., 1813.}	105.595	0.661112	newly 5 weeks cumul	9330.	22 524.8	{4013., 67 847.}	161.842	0.306283
14	newly 3 weeks spec	2628.	826.49	{77., 2111.}	72.7005	0.811222	newly 3 weeks cumul	11 099.	17 548.4	{2486., 37 600.}	109.937	0.391428	newly 5 weeks spec	1313.	63.78	{2., 222.}	95.1424	1.8612	newly 5 weeks cumul	14 282.	17 835.4	{2512., 38 484.}	88.5581	0.398973
15	newly 3 weeks spec	1421.	1799.9	{300., 6253.}	112.926	0.470085	newly 3 weeks cumul	6948.	9904.25	{3923., 23 659.}	85.4449	0.258858	newly 5 weeks spec	621.	512.32	{37., 1297.}	107.659	0.805459	newly 5 weeks cumul	8459.	11 374.8	{4102., 36 222.}	86.8337	0.287606
16	newly 3 weeks spec	1685.	2504.98	{0., 7531.}	150.541	0.967471	newly 3 weeks cumul	8356.	8439.31	{938., 20 224.}	85.6009	0.447929	newly 5 weeks spec	644.	1781.09	{0., 5769.}	272.536	0.967923	newly 5 weeks cumul	10 017.	12 433.4	{1277., 32 895.}	108.77	0.466986
17	newly 3 weeks spec	244.	1424.3	{256., 2681.}	495.926	0.583802	newly 3 weeks cumul	2213.	7185.73	{1000., 15 369.}	243.987	0.431128	newly 5 weeks spec	41.	928.15	{38., 955.}	2172.37	0.873673	newly 5 weeks cumul	2358.	9237.37	{1469., 20 179.}	308.327	0.474178
18	newly 3 weeks spec	946.	3426.02	{180., 13 123.}	317.161	0.493836	newly 3 weeks cumul	4981.	20 354.8	{2064., 61 610.}	335.247	0.493911	newly 5 weeks spec	314.	929.89	{21., 3298.}	289.443	0.688023	newly 5 weeks cumul	5835.	23 071.9	{2219., 71 409.}	323.319	0.483151
19	newly 3 weeks spec	2246.	2019.85	{70., 5485.}	109.018	0.855236	newly 3 weeks cumul	9933.	8244.82	{475., 24 610.}	93.763	0.66289	newly 5 weeks spec	1136.	1066.23	{2., 3262.}	114.258	1.12484	newly 5 weeks cumul	12 584.	10 752.2	{624., 28 057.}	96.699	0.683478
20	newly 3 weeks spec	688.	2886.18	{185., 9513.}	360.974	0.472493	newly 3 weeks cumul	3837.	16 256.5	{1881., 28 714.}	350.561	0.497531	newly 5 weeks spec	219.	1120.78	{19., 4156.}	479.708	0.627531	newly 5 weeks cumul	4426.	19 216.2	{2003., 44 241.}	361.315	0.498564
21	newly 3 weeks spec	566.	763.28	{130., 1536.}	90.3922	0.344054	newly 3 weeks cumul	3401.	4881.77	{1274., 13 389.}	80.4813	0.260651	newly 5 weeks spec	156.	287.08	{36., 547.}	154.295	0.53851	newly 5 weeks cumul	3828.	5625.91	{1448., 14 138.}	81.2834	0.252923
22	newly 3 weeks spec	1150.	732.65	{35., 1737.}	86.4983	0.734873	newly 3 weeks cumul	5834.	14 132.4	{1706., 36 589.}	170.556	0.375334	newly 5 weeks spec	400.	72.05	{0., 220.}	90.8025	1.67563	newly 5 weeks cumul	6931.	14 418.1	{1707., 36 968.}	141.224	0.349549
23	newly 3 weeks spec	361.	3613.85	{133., 14 698.}	924.867	0.734674	newly 3 weeks cumul	2348.	16 992.7	{697., 57 149.}	646.888	0.639321	newly 5 weeks spec	79.	1811.9	{17., 6468.}	2217.39	1.00041	newly 5 weeks cumul	2588.	21 325.7	{996., 75 019.}	745.627	0.679884
24	newly 3 weeks spec	617.	3150.26	{375., 8055.}	429.9	0.454359	newly 3 weeks cumul	3939.	16 497.4	{2679., 33 298.}	331.924	0.452919	newly 5 weeks spec	171.	1197.8	{39., 4046.}	645.848	0.599364	newly 5 weeks cumul	4404.	19 668.6	{2998., 52 309.}	358.411	0.465837
25	newly 3 weeks spec	1330.	2449.49	{69., 4176.}	201.895	0.758175	newly 3 weeks cumul	7007.	9669.57	{514., 25 092.}	149.214	0.670307	newly 5 weeks spec	443.	842.23	{12., 1298.}	208.53	0.818658	newly 5 weeks cumul	8227.	12 010.8	{565., 29 168.}	157.8	0.679305
26	newly 3 weeks spec	920.	2740.24	{133., 7581.}	240.867	0.515797	newly 3 weeks cumul	5136.	11 543.3	{1104., 26 654.}	169.295	0.419596	newly 5 weeks spec	345.	1390.59	{18., 3894.}	345.354	0.704702	newly 5 weeks cumul	6057.	14 857.6	{1351., 40 201.}	186.359	0.4403
27	newly 3 weeks spec	2118.	855.36	{245., 1384.}	64.8074	0.519216	newly 3 weeks cumul	9250.	9873.86	{2727., 17 260.}	82.8798	0.356072	newly 5 weeks spec	1079.	199.27	{45., 398.}	81.532	0.987698	newly 5 weeks cumul	11 803.	10 456.5	{3055., 17 609.}	70.7175	0.353576
28	newly 3 weeks spec	196.	673.41	{142., 1144.}	256.434	0.483024	newly 3 weeks cumul	1632.	6139.42	{1687., 6316.}	284.531	0.411589	newly 5 weeks spec	32.	204.19	{7., 464.}	556.531	0.72741	newly 5 weeks cumul	1750.	6691.81	{1991., 8752.}	288.755	0.42633
29	newly 3 weeks spec	1912.	1654.79	{117., 4139.}	98.1104	0.655404	newly 3 weeks cumul	9043.	19 049.3	{2550., 49 630.}	155.401	0.407551	newly 5 weeks spec	849.	197.44	{3., 544.}	86.7962	1.3472	newly 5 weeks cumul	11 162.	19 801.4	{2715., 51 396.}	130.19	0.403953
30	newly 3 weeks spec	1221.	629.95	{51., 1382.}	70.2793	0.653617	newly 3 weeks cumul	6812.	4558.75	{899., 7179.}	56.3437	0.418229	newly 5 weeks spec	428.	299.77	{0., 696.}	86.1612	1.13269	newly 5 weeks cumul	7969.	5280.44	{985., 8189.}	56.6932	0.418505
31	newly 3 weeks spec	1586.	2647.15	{83., 4777.}	116.071	0.52616	newly 3 weeks cumul	7252.	32 358.6	{8594., 80 455.}	356.114	0.528031	newly 5 weeks spec	689.	482.96	{14., 1240.}	99.7068	0.920287	newly 5 weeks cumul	9024.	33 874.	{9566., 81 397.}	285.415	0.466672
32	newly 3 weeks spec	2184.	1144.72	{101., 2728.}	87.0925	0.748378	newly 3 weeks cumul	9583.	6042.82	{828., 11 214.}	76.5718	0.563197	newly 5 weeks spec	1094.	449.27	{21., 1472.}	91.4963	1.02933	newly 5 weeks cumul	12 124.	7202.19	{885., 14 645.}	79.3599	0.607751
33	newly 3 weeks spec	643.	562.41	{67., 695.}	70.776	0.395666	newly 3 weeks cumul	3804.	4100.19	{1357., 4831.}	66.9282	0.259887	newly 5 weeks spec	197.	199.12	{4., 371.}	98.9746	0.626999	newly 5 weeks cumul	4382.	4624.94	{1451., 5170.}	66.6194	0.2631
34	newly 3 weeks spec	883.	2018.64	{39., 6793.}	212.915	0.653773	newly 3 weeks cumul	4731.	16 852.8	{2425., 49 668.}	277.031	0.435213	newly 5 weeks spec	278.	577.84	{1., 1101.}	239.755	1.34442	newly 5 weeks cumul	5501.	18 472.	{2435., 53 299.}	260.312	0.424862
35	newly 3 weeks spec	239.	945.46	{222., 1004.}	306.017	0.412967	newly 3 weeks cumul	1921.	4396.5	{1484., 5156.}	141.67	0.318159	newly 5 weeks spec	50.	552.51	{33., 469.}	1023.46	0.640665	newly 5 weeks cumul	2064.	5669.91	{1915., 6271.}	184.671	0.343957
36	newly 3 weeks spec	2432.	1978.66	{69., 4716.}	107.975	0.824492	newly 3 weeks cumul	10 891.	10 336.	{524., 22 184.}	104.176	0.637859	newly 5 weeks spec	1211.	637.18	{14., 2104.}	94.9777	1.08857	newly 5 weeks cumul	13 782.	12 098.1	{577., 27 682.}	102.626	0.680089
37	newly 3 weeks spec	1023.	2623.09	{121., 7173.}	194.009	0.496063	newly 3 weeks cumul	5532.	38 859.5	{1558., 116 577.}	620.447	0.705919	newly 5 weeks spec	383.	239.32	{8., 446.}	75.7963	0.72258	newly 5 weeks cumul	6497.	39 802.2	{2399., 118 364.}	530.356	0.659881
38	newly 3 weeks spec	915.	3128.67	{74., 13 758.}	309.793	0.543459	newly 3 weeks cumul	5124.	10 969.7	{399., 39 031.}	187.205	0.510103	newly 5 weeks spec	323.	1864.56	{37., 10 278.}	540.508	0.593948	newly 5 weeks cumul	5962.	15 323.8	{503., 58 069.}	227.077	0.522308
39	newly 3 weeks spec	1339.	1177.99	{100., 1975.}	86.0441	0.511821	newly 3 weeks cumul	6703.	6873.52	{791., 15 348.}	86.8638	0.421657	newly 5 weeks spec	464.	415.33	{15., 634.}	99.8728	0.677499	newly 5 weeks cumul	7906.	7986.13	{1012., 16 365.}	86.2437	0.428583
40	newly 3 weeks spec	1822.	2115.9	{191., 5566.}	92.3502	0.421285	newly 3 weeks cumul	8114.	15 887.5	{2406., 27 827.}	121.099	0.264916	newly 5 weeks spec	874.	647.3	{14., 1288.}	97.7391	0.748899	newly 5 weeks cumul	10 273.	17 660.8	{2866., 46 937.}	109.158	0.278089
41	newly 3 weeks spec	362.	1121.85	{180., 2114.}	228.251	0.391364	newly 3 weeks cumul	2774.	6239.7	{861., 8025.}	153.618	0.345171	newly 5 weeks spec	74.	459.01	{59., 950.}	529.797	0.56633	newly 5 weeks cumul	2996.	7399.85	{1082., 10 911.}	171.774	0.357618
42	newly 3 weeks spec	2612.	3203.08	{112., 10 460.}	107.591	0.614012	newly 3 weeks cumul	10 679.	25 379.2	{2159., 91 014.}	179.03	0.415929	newly 5 weeks spec	1374.	802.88	{1., 2763.}	81.444	1.1306	newly 5 weeks cumul	13 825.	27 732.8	{2347., 96 874.}	149.122	0.412278
43	newly 3 weeks spec	2253.	1574.41	{94., 4154.}	81.9951	0.676321	newly 3 weeks cumul	10 165.	7722.27	{852., 20 351.}	78.7161	0.544268	newly 5 weeks spec	1018.	798.13	{16., 2270.}	103.288	0.903823	newly 5 weeks cumul	12 779.	9645.02	{906., 29 019.}	80.1681	0.575168
44	newly 3 weeks spec	1384.	910.22	{68., 2964.}	82.0824	0.670156	newly 3 weeks cumul	6755.	16 861.7	{2991., 44 452.}	176.226	0.364891	newly 5 weeks spec	626.	120.03	{1., 309.}	89.1358	1.62072	newly 5 weeks cumul	8295.	17 290.9	{3011., 47 039.}	140.722	0.335045
45	newly 3 weeks spec	1196.	1533.55	{0., 2376.}	142.258	1.99199	newly 3 weeks cumul	6409.	17 811.5	{1762., 59 725.}	218.448	0.463265	newly 5 weeks spec	536.	743.98	{0., 914.}	157.011	1.91253	newly 5 weeks cumul	7757.	19 658.9	{1995., 72 817.}	193.726	0.439351
46	newly 3 weeks spec	1149.	678.95	{39., 1654.}	81.2889	0.722399	newly 3 weeks cumul	6165.	6721.06	{930., 10 313.}	94.921	0.410509	newly 5 weeks spec	377.	254.92	{0., 583.}	100.313	1.21024	newly 5 weeks cumul	7203.	7363.34	{935., 10 596.}	91.5488	0.426178
47	newly 3 weeks spec	1358.	1295.57	{53., 1956.}	115.191	0.688257	newly 3 weeks cumul	7212.	5085.97	{483., 11 609.}	90.8285	0.618475	newly 5 weeks spec	550.	1149.61	{9., 1044.}	227.773	0.875546	newly 5 weeks cumul	8640.	7475.36	{605., 13 693.}	104.484	0.625406
48	newly 3 weeks spec	874.	2045.43	{0., 4487.}	234.283	2.01806	newly 3 weeks cumul	5250.	20 191.3	{2023., 71 234.}	304.937	0.429414	newly 5 weeks spec	248.	1028.59	{0., 947.}	418.867	1.84555	newly 5 weeks cumul	5990.	22 721.5	{2023., 75 394.}	301.064	0.437521
49	newly 3 weeks spec	879.	2341.13	{36., 8056.}	265.334	0.746849	newly 3 weeks cumul	4780.	22 242.2	{3267., 70 186.}	378.028	0.46198	newly 5 weeks spec	321.	513.34	{0., 1661.}	198.953	1.59181	newly 5 weeks cumul	5641.	23 810.5	{3691., 75 114.}	336.527	0.424233
50	newly 3 weeks spec	1513.	1954.3	{159., 4668.}	82.2327	0.35779	newly 3 weeks cumul	7746.	14 212.9	{1832., 25 081.}	111.6	0.313313	newly 5 weeks spec	636.	553.12	{22., 1257.}	86.478	0.562001	newly 5 weeks cumul	9438.	15 778.8	{2000., 31 664.}	100.658	0.311353

1	newly 47 weeks cumul	7014.	17 293.9	{8144., 48 707.}	155.609	0.369539
2	newly 54 weeks cumul	6291.	21 029.	{5320., 67 553.}	247.013	0.383551
3	newly 56 weeks cumul	8546.	9341.49	{1155., 21 198.}	79.8817	0.347386
4	newly 54 weeks cumul	8186.	16 558.	{2441., 35 566.}	136.216	0.355724
5	newly 56 weeks cumul	2676.	5698.94	{1735., 10 682.}	132.476	0.264314
6	newly 55 weeks cumul	1849.	22 093.8	{2152., 77 572.}	1099.71	0.669758
7	newly 47 weeks cumul	13 516.	21 646.9	{8590., 58 658.}	84.4809	0.217787
8	newly 54 weeks cumul	8594.	43 764.4	{7155., 107 219.}	422.014	0.572142
9	newly 56 weeks cumul	2718.	9497.02	{2691., 21 362.}	256.333	0.323329
10	newly 52 weeks cumul	16 196.	21 980.9	{2075., 79 345.}	104.213	0.411325
11	newly 51 weeks cumul	13 920.	21 602.1	{903., 85 509.}	145.191	0.59623
12	newly 52 weeks cumul	8581.	29 760.	{5286., 97 948.}	265.101	0.384954
13	newly 53 weeks cumul	10 733.	23 393.	{4252., 78 896.}	146.003	0.303569
14	newly 46 weeks cumul	17 443.	17 866.7	{2512., 38 493.}	78.1742	0.424146
15	newly 47 weeks cumul	9505.	12 012.3	{4127., 39 567.}	86.8233	0.312507
16	newly 50 weeks cumul	11 020.	17 116.6	{1664., 55 871.}	136.479	0.477188
17	newly 56 weeks cumul	2381.	12 720.6	{2500., 21 796.}	447.599	0.53134
18	newly 55 weeks cumul	6316.	24 643.	{2235., 79 667.}	318.81	0.478249
19	newly 50 weeks cumul	14 784.	15 291.	{696., 39 382.}	111.962	0.711306
20	newly 55 weeks cumul	4693.	20 994.5	{2035., 65 604.}	374.261	0.501624
21	newly 56 weeks cumul	3988.	6401.5	{1728., 16 218.}	92.6605	0.268425
22	newly 52 weeks cumul	7573.	14 460.8	{1707., 36 988.}	129.381	0.348234
23	newly 53 weeks cumul	2670.	26 389.9	{1486., 102 048.}	906.033	0.726778
24	newly 55 weeks cumul	4602.	22 421.1	{3396., 72 205.}	398.667	0.491028
25	newly 51 weeks cumul	8870.	13 035.5	{578., 29 244.}	157.884	0.677903
26	newly 49 weeks cumul	6590.	20 067.4	{1493., 60 346.}	241.021	0.507265
27	newly 49 weeks cumul	14 480.	10 773.4	{3055., 17 638.}	66.4621	0.378165
28	newly 56 weeks cumul	1781.	7198.47	{2368., 9718.}	308.538	0.454998
29	newly 53 weeks cumul	12 786.	19 982.3	{2715., 51 561.}	114.418	0.403122
30	newly 56 weeks cumul	8592.	6212.91	{986., 9776.}	59.3677	0.411703
31	newly 46 weeks cumul	10 290.	34 787.	{9772., 81 829.}	248.506	0.430234
32	newly 52 weeks cumul	14 626.	8006.77	{933., 18 134.}	81.4512	0.658019
33	newly 56 weeks cumul	4693.	5045.06	{1647., 6119.}	66.5723	0.257794
34	newly 52 weeks cumul	5915.	19 245.9	{2435., 58 419.}	251.805	0.419098
35	newly 56 weeks cumul	2096.	7283.16	{2314., 6988.}	255.474	0.387061
36	newly 52 weeks cumul	16 479.	12 989.	{591., 32 014.}	98.6807	0.720843
37	newly 53 weeks cumul	7036.	40 433.4	{2400., 118 437.}	493.575	0.647054
38	newly 50 weeks cumul	6391.	19 265.1	{582., 93 220.}	267.903	0.530311
39	newly 52 weeks cumul	8666.	8669.38	{1023., 16 460.}	85.4953	0.43213
40	newly 53 weeks cumul	12 291.	19 084.7	{2973., 67 164.}	105.58	0.31084
41	newly 56 weeks cumul	3059.	8575.33	{1290., 15 655.}	201.333	0.382029
42	newly 47 weeks cumul	17 141.	28 756.8	{2726., 98 333.}	124.756	0.411285
43	newly 52 weeks cumul	15 131.	11 347.5	{918., 30 376.}	81.8059	0.607772
44	newly 52 weeks cumul	9359.	17 402.8	{3011., 47 501.}	124.846	0.332245
45	newly 54 weeks cumul	8640.	22 458.1	{4006., 76 775.}	194.099	0.436423
46	newly 56 weeks cumul	7768.	7960.19	{935., 10 956.}	92.3382	0.432698
47	newly 51 weeks cumul	9536.	11 169.5	{657., 18 192.}	122.846	0.598269
48	newly 54 weeks cumul	6323.	25 152.6	{2023., 82 313.}	320.477	0.461112
49	newly 52 weeks cumul	6148.	24 347.1	{3691., 77 685.}	311.209	0.402115
50	newly 53 weeks cumul	10 556.	16 577.2	{2056., 35 610.}	95.5202	0.314551
